# Saliva cotinine concentrations in pregnant women who smoke and use nicotine patches

**DOI:** 10.1111/add.14662

**Published:** 2019-06-30

**Authors:** Ravinder Claire, Tim Coleman, Jo Leonardi‐Bee, Ivan Berlin

**Affiliations:** ^1^ Division of Primary Care University of Nottingham Nottingham UK; ^2^ Division of Epidemiology and Public Health University of Nottingham Nottingham UK; ^3^ Département de pharmacologie Hôpital Pitié‐Salpêtrière Paris France; ^4^ Centre Universitaire de Médecine Générale et Santé publique, Centre Hospitalier Universitaire Vaudois Lausanne Switzerland; ^5^ CESP‐INSERM U1018 Paris France

**Keywords:** Cotinine, nicotine, nicotine replacement therapy, pregnancy, smoking, smoking cessation

## Abstract

**Background and Aims:**

Due to concerns about increased exposure to nicotine, pregnant women using nicotine replacement therapy (NRT) to stop smoking are usually advised to stop using NRT if they relapse to smoking. This study investigated whether this is justified. We compared changes in saliva cotinine from baseline to 2 weeks post‐target quit date pregnant smokers who relapsed to smoking and continued to use their patches having been assigned to use nicotine patches or placebo.

**Design and Setting:**

Controlled pre–post design stratified by intervention condition from the ‘Study of Nicotine Patch in Pregnancy’, a randomized, placebo‐controlled trial.

**Participants:**

A sample of 268 pregnant women, assigned placebo (*n* = 122) or nicotine (*n* = 146) patches, who returned for further supplies of patches and who reported any smoking in the week prior to a visit at 2 weeks after their target quit date.

**Measurements:**

Saliva cotinine concentrations were measured at baseline and 2 weeks after participants’ target quit dates. Any smoking in the previous week was assessed by self‐report, validated by expired air carbon monoxide (CO).

**Findings:**

There was no change in saliva cotinine concentrations between baseline and 2 weeks post‐target quit date in saliva cotinine concentration in the nicotine patch group [ratio of geometric means = 0.94, 95% confidence interval (CI) = 0.83 to 1.07; *P* = 0.37, Bayes factor = 0.15]. However, there was a reduction in reported number of cigarettes smoked/day (mean difference −6, 95% CIs −7 to −5, *P* < 0.001) and in CO concentrations (mean difference −3.0 parts per million, 95% CIs −4.2 to −1.9, *P* < 0.001). These changes were not significantly different from changes in the placebo group except for cigarette consumption, which reduced more in the nicotine group (*P* = 0.046).

**Conclusions:**

In women trying to stop smoking with the aid of a nicotine patch but having smoked at 2 weeks post‐target quit, their nicotine concentration did not change from baseline, but they reported smoking fewer cigarettes and had lower carbon monoxide concentrations.

## Introduction

Smoking during pregnancy is the leading modifiable risk factor for poor maternal and infant health outcomes. Pregnancy‐related health problems associated with smoking during pregnancy include complications during labour, increased risk of miscarriage, premature birth, stillbirth and low birth weight 1, 2, 3. Despite this, approximately 12% of pregnant women in the United Kingdom, 13% in the United States and 20% in France continue to smoke during pregnancy 4, 5, 6. Several national guidelines have adopted using nicotine replacement therapy (NRT) for supporting pregnant smokers to quit, based on the idea that NRT is probably safer than smoking as it does not contain the toxins present in tobacco smoke 7, 8.

While NRT has been proven to be effective in non‐pregnant smokers 9, its efficacy in pregnancy is uncertain 10. The reason for this uncertainty is unclear; however, it is hypothesized that physiological changes in pregnancy could affect nicotine's metabolism 11. Potential factors for the increased metabolism rate include a higher concentration or activity of metabolic enzymes involved and increased blood flow through the liver during pregnancy 12. Cotinine is the principal metabolite of nicotine, and the clearance of nicotine and cotinine is 60 and 140% higher, respectively, during pregnancy 13. An increase in metabolic rate could signify that nicotine supplied through standard dose NRT may be insufficient to alleviate smoking withdrawal symptoms in pregnancy and to provide therapeutic effects.

A systematic review and meta‐analysis comparing nicotine exposure in pregnant women when smoking, and their nicotine exposure when abstinent and using NRT, found that NRT exposes women to lower doses of nicotine than does smoking 14. Generally, in studies included in this review, such as the Smoking, Nicotine and Pregnancy (SNAP) trial, women were instructed to discontinue use of nicotine patches if they had even brief smoking lapses 15. This mimics routine health care, where pregnant women are usually advised to stop using NRT if they lapse to smoking, even for short periods. There is concern that concomitant smoking and NRT use could increase exposure to nicotine and potentially more tobacco smoke toxins if they smoked heavily when using NRT. However, in pregnancy this assumption is untested, and we know little about women's smoking behaviour when they use NRT concurrently. This is important, as women who lapse to smoking may still want to quit. In a non‐pregnant population, continued use of nicotine patches has been found to promote recovery from lapses 16; if this is the case during pregnancy, women may have better chances of cessation if NRT is continued.

This study aims to investigate and compare: (1) changes in saliva cotinine and other indicators of smoking intensity in women using nicotine or placebo patches and smoking concurrently with those when they only smoked; and (2) whether these changes differed between nicotine and placebo patch use.

## Methods

### Design

This is a secondary analysis of data from the Study of Nicotine Patch in Pregnancy (SNIPP) 17. SNIPP was a multi‐centre, double‐blind, randomized, placebo‐controlled study conducted in France using 16‐hour nicotine patches. The trial randomized 402 women to either nicotine (*n* = 203) or placebo patches (*n* = 199). The study was approved by the Ethics Committee of the Pitié‐Salpêtrière Hospital, Paris, France.

### Participants

Participants were eligible for inclusion in the SNIPP trial if they smoked at least five cigarettes per day, were aged more than 18 years, of 12–20 weeks’ gestation and scored at least 5 on a scale measuring motivation to stop smoking (range 0–10) 17. Prior to enrolment, participants attended a baseline visit where demographic, obstetric, physiological characteristics and smoking behaviour data were collected, and saliva cotinine concentrations were determined. At this stage, participants were given 2 weeks to quit smoking or reduce the number of cigarettes to fewer than five a day. If after this 2‐week period they were unable to do either of these they could be randomized, receive the study drug and set a quit date when treatment began. Participants were asked to stop smoking on a pre‐defined quit date and were randomized to either placebo or nicotine patches. Participants were told that they could continue using nicotine patches during smoking lapses. Moreover, patch doses were adjusted according to the previous saliva cotinine determination to optimize the nicotine substitution; this resulted in participants receiving a mean nicotine dose of 18 mg/day [standard deviation (SD) = 6.8] in the nicotine patch arm.

### Measures

In the SNIPP trial, abstinence was defined as self‐reported abstinence, confirmed by expired air carbon monoxide (CO) concentration ≤ 8 parts per million (p.p.m.) (Smokeanalyzer^®^; Bedfont Scientific Ltd, Rochester, Kent, UK) 18. Saliva cotinine samples were collected by placing a cotton roll in the gingival cleft for 1 minute, which was then placed immediately into a Salivette tube (Sarstedt, Nümbrecht, Germany) 18. Samples were kept at 4°C and were sent to the central biochemistry laboratory (Hôpital Pitié‐Salpêtrière, Laboratoire de Biochimie, Dr N. Jacob) within 24 hours for determination 18. The quantification limit for cotinine was 7.5 mg/l and the between‐run coefficient of variation 5–8% 18.

**Figure 1:**
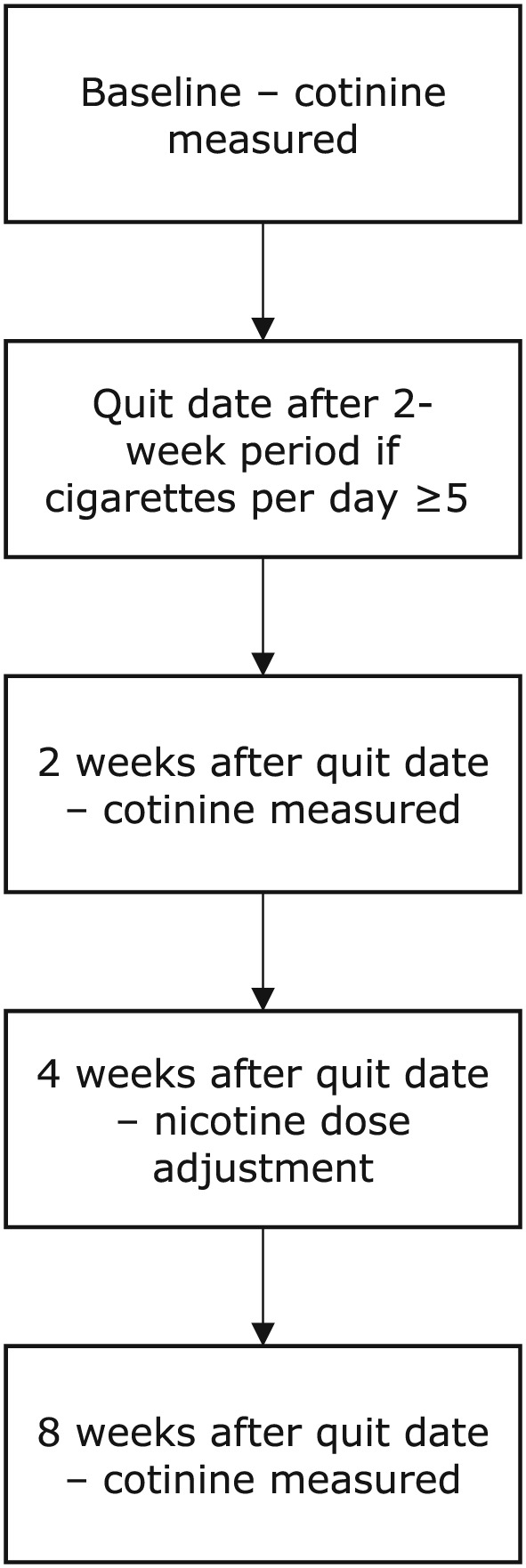
Flow‐chart to show each planned visit in the ‘Study of Nicotine Patch in Pregnancy’ relevant to the current study

Figure 1 shows when trial visits occurred and measurements were made. Saliva cotinine concentrations were determined at baseline, 2 weeks after quit date and 8 weeks after quit date, with nicotine doses adjusted after each of these visits at 4 and 12 weeks after quit date, respectively. Nicotine doses were adjusted using a conversion factor of 0.1. For example, a saliva cotinine concentration of 100 ng/l equated to a prescription of one 10‐mg patch 17. At baseline, body mass index (BMI), gestational age, ethnicity and Fagerström Test for Cigarette Dependence (FTCD) scores were recorded. As well as at baseline, at each visit women reported any smoking in the previous week, validated by expired air CO. Additionally, intensity of craving for tobacco via the French Tobacco Craving Questionnaire, 12 items (FTCQ‐12) and the number of cigarettes smoked by the participant in the last week were assessed. The SNIPP trial recorded cigarette consumption in the past week, rather than cigarettes per day, due to large day‐to‐day fluctuations in cigarette consumption 19, 20. Partner smoking in the previous week was also assessed, as the second‐hand smoke exposure is likely to increase cotinine measures. Women were permitted to use nicotine patches from quit date until delivery. A more extensive description is available elsewhere 17.

In this study we used data from women collected at 2 weeks after the quit date who had been allocated nicotine or placebo patches but who reported any smoking in the previous week. A second sample of data collected at 8 weeks after the quit date from women who had smoked in the previous week were used as a sensitivity analysis. Not all women who had cotinine measured at 2 weeks returned for the 8‐week visit, and 8‐week data also included women who did not return at 2 weeks. We selected women from 2 weeks rather than 8 weeks after the quit date for the main analysis, as this time‐point was earlier in gestation, so nicotine metabolism changes since the baseline visit would probably be small and have less impact on findings 21.

### Analyses

For baseline data, continuous measures were reported as means with SDs, and categorical measures were reported using frequencies and percentages. Participant and partner's smoking in the previous week were divided by 7 to achieve cigarettes smoked per day. *T*‐tests were used to assess whether there were any systematic differences in baseline characteristics between women who were included and those excluded from this study. We used a natural log‐transformation of salivary cotinine concentrations to achieve a normal distribution.

For both nicotine and placebo patch groups we used paired *t*‐tests to assess ‘within‐participant’ differences between cotinine, CO, cravings, number of cigarettes smoked by the participant and number of cigarettes smoked by their partner, measured at baseline and at 2 weeks. The same analyses were conducted using data from 8 weeks. For saliva cotinine, we present the back‐transformed estimates of treatment differences, which is the ratio of the geometric means. Next, we used linear regression analysis to test for an interaction between the measures mentioned above and nicotine patch assignment. We then aimed to identify whether the interactions were significant at increasing increments of baseline values in cotinine, CO, cravings, number of cigarettes smoked by the participant and number of cigarettes smoked by their partner. Findings are presented graphically. *P*‐values less than 0.05 were deemed statistically significant. All analyses were conducted using Stata version 15.

After undertaking the planned analyses we generated a Bayes factor from the difference in saliva cotinine, using an online calculator 22. We were unable to identify any studies that investigated nicotine intake of concurrent smokers and NRT users in pregnancy, so an expected difference of 139.3 ng/ml was taken from a study of nicotine intake outside pregnancy 23. We used a conservative approach for estimation using a half‐normal distribution, where the standard deviation is equal to the expected effect size.

## Results

In the SNIPP trial, 203 women were assigned to the nicotine patch arm and 199 women were assigned to the placebo patch arm. At 2 weeks after the quit date, 167 (82.3%) and 148 (74.4%) women returned for the visit in the nicotine patch and placebo patch arms, respectively. In the nicotine patch arm, 149 (73.4%) had smoked in the week prior to the visit and 18 (8.9%) were abstinent whereas, in the placebo group, 131 (65.8%) had smoked during the week prior to the visit and 17 (8.5%) were abstinent. Overall, 12 women had missing cotinine data at this point and were excluded from the study, leaving a sample of 268 for analysis (146 in the nicotine group and 122 in the placebo group).

When comparing SNIPP trial participants excluded from this study with those included, it was found that more women in this study had a partner who smoked. Table 1 gives baseline characteristics of women in both study groups and, using these descriptors, both groups were broadly similar. From the participants who provided 2‐week data, those assigned nicotine patch had a mean age of 30 years and gestational age at baseline of 12.8 weeks; therefore, their mean gestational age at 2 weeks post‐quit date would be between 16 and 17 weeks.

**Table 1:** Participant baseline characteristics; n (%) or mean (standard deviation).

Characteristic	Women on nicotine patch (n = 146)	Women on placebo patch (n = 122)
Age (years)	29.70 (6.00)	28.88 (5.03)
BMI (kg/m^2^)	25.52 (5.40)	25.21 (5.33)
Gestational age at baseline (weeks)	12.8 (3.2)	12.6 (5.4)
Ethnicity		
European	139 (95)	115 (94)
African	4 (3)	4 (3)
Asian	1 (1)	1 (1)
Other	2 (1)	2 (2)
Current cigarettes smoking per day		
5–10	66 (45)	55 (45)
11–20	69 (47)	50 (41)
21–30	7 (5)	16 (13)
>30	4 (3)	1 (1)
Fagerström Test for Cigarette Dependence^a^		
Very low	32 (22)	20 (16)
Low	34 (23)	42 (34)
Medium	29 (20)	18 (15)
High	43 (29)	33 (27)
Very high	8 (6)	9 (7)
Partner smoking		
Yes	99 (69)	90 (75)
Saliva cotinine (ng/ml)	143.86 (82.81)	144.36 (74.33)
Expired air carbon monoxide (p.p.m.)	11.8 (6.7)	12.2 (7.3)
French Tobacco Craving Questionnaire score	33.64 (8.60)	35.55 (9.53)

a Fagerström Test for Cigarette Dependence is a six‐item test where answers are summed to yield a total score of 0–10. The higher the total score, the more intense is the patient's physical dependence on cigarettes; i.e. a score between 0–2 indicates a very low level of dependence on cigarettes, and 8–10 indicates a very high‐level dependence on cigarettes 24. BMI = body mass index; p.p.m. = parts per million.

Table 2 compares indicators of smoking intensity between baseline and 2 weeks after the quit date for pregnant smokers in both the placebo and nicotine patch groups. In the nicotine group there was no significant difference between cotinine concentrations [ratio of geometric means = 0.94 ng/ml, 95% confidence interval (CI) = 0.83–1.07 ng/ml; *P* = 0.37, Bayes factor = 0.15], but CO concentrations significantly decreased from baseline to 2 weeks after the quit date (mean difference − 3.0 p.p.m., 95% CI = −4.2 to −1.9 p.p.m.; *P* < 0.001), whereas the placebo group exhibited a significant reduction in cotinine (ratio of geometric means = 0.68 ng/ml, 95% CI = 0.59–0.78 ng/ml; *P* < 0.001) as well as a reduction in CO concentration (mean difference = −2.0 p.p.m., 95% CI = −3.8 to −0.2 p.p.m., *P* < 0.028). There were also significantly lower levels of craving, lower numbers of cigarettes smoked in the previous week and women's partners were reported to have smoked fewer cigarettes in both nicotine and placebo patch groups.

**Table 2:** Baseline to 2 weeks after the quit date ‘within‐participant’ differences in indicators of smoking intensity in pregnant smokers by treatment group, with a significance test for interaction with nicotine patch.

Characteristic	Nicotine patch (n = 146)				Placebo patch (n = 122)				Interaction P‐value^a^
	Baseline mean (SD)	2 weeks after quit date mean (SD)	Mean difference (95% CI)	P‐value^b^	Baseline mean (SD)	2 weeks after quit date mean (SD)	Mean difference (95% CI)	P‐value^c^	
Saliva cotinine^e^ (ng/ml)	117.83	111.14	0.94 (0.83 to 1.07)	0.370	122.46	83.01	0.68 (0.59 to 0.78)	< 0.001	0.148
Expired air carbon monoxide (p.p.m.)	11.8 (6.7)	8.7 (6.5)	−3.0 (−4.2 to −1.9)	< 0.001	12.2 (7.3)	10.2 (9.1)	−2.0 (−3.8 to −0.2)	0.028	0.498
FTCQ‐12^d^	33.75 (8.63)	31.38 (8.06)	−2.38 (−3.88 to −0.87)	0.002	35.84 (9.00)	33.36 (8.57)	−2.49 (−4.37 to −0.60)	0.010	0.317
Number of cigarettes smoked per day	12 (6)	6 (5)	−6 (−7 to −5)	< 0.001	12 (6)	6 (6)	−6 (−7 to −5)	< 0.001	0.046
Number of cigarettes partner smoked per day	17 (9)	15 (7)	−1 (−2 to 0)	0.026	16 (7)	14 (7)	−2 (−3 to −1)	0.003	0.168

Paired *t*‐tests were used to compare differences at baseline and 2 weeks after the quit date. A linear model was used to test for an interaction of nicotine patch between baseline and 2 weeks. SD = standard deviation; CI = confidence interval; p.p.m. = parts per million.

a *P*‐value for interaction of nicotine patch with indicators of smoking intensity at baseline compared with at 2 weeks after the quit date.

b *P*‐value for the difference between indicators of smoking intensity between baseline and 2 weeks in the nicotine patch group.

c *P*‐value for the difference between indicators of smoking intensity between baseline and 2 weeks in the placebo patch group.

d FTCQ−12 = French Tobacco Craving Questionnaire score.

e Back‐transformed saliva cotinine data. Means represent geometric means. Mean difference presented as ratio of geometric means.

**Figure 2:**
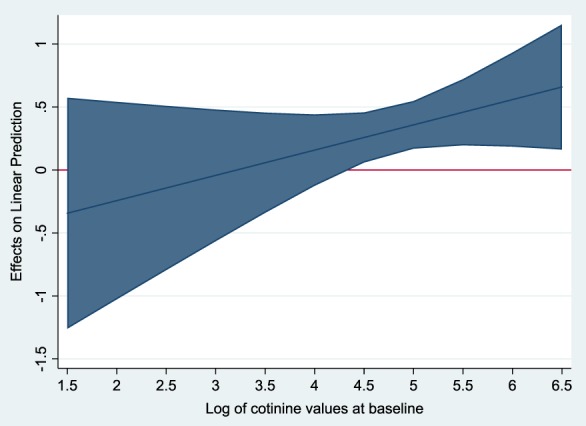
Graph to show interaction of nicotine patches on cotinine concentrations at 2 weeks with increasing baseline cotinine concentrations. The shaded area represents the 95% confidence intervals. As the shaded area for log cotinine > 4.5 is above 0, there is a significant interaction of nicotine patches for an increase in cotinine at 2 weeks in women with log cotinine concentrations of greater than 4.5 (back‐transformed to 90 ng/ml), compared with placebo. [Colour figure can be viewed at wileyonlinelibrary.com]

Table 2 also reports results for interaction tests between the indicators of smoking intensity and nicotine patch assignment. There was a significant interaction between nicotine patch assignment and a reduction in number of cigarettes smoked (*P* = 0.046). This means that women assigned nicotine patches smoked less at week 2 compared to women assigned placebo patches. Interactions between the remaining indicators of smoking intensity and nicotine patch assignment were not significant. Upon further exploration it was discovered that there was an interaction between nicotine patch assignment and women with higher baseline cotinine concentrations (Fig. 2). Women assigned nicotine patches with baseline saliva cotinine concentrations of approximately 90 ng/ml and above had higher cotinine concentrations at week 2 compared to women assigned placebo patches.

In the sensitivity analysis, the 8‐week data showed a similar pattern to the 2‐week data (Supporting information, Table S1). There was no significant difference between cotinine concentrations at baseline and 8 weeks in the nicotine patch group (ratio of geometric means = 0.85 ng/ml, 95% CI = 0.71–1.00 ng/ml; *P* = 0.055, Bayes factor = 0.12); however, there were significant reductions for all other indicators of smoking intensity aside from craving score (mean difference = −1.69, 95% CI = −3.58 to 0.20 *P* = 0.079). In women assigned placebo patches, there were significant reductions for all indicators of smoking cessation aside from expired CO concentration (mean difference − 2.4 p.p.m., 95% CI = − 5.0 to 0.3 p.p.m., *P* < 0.077). The interaction tests found no significant interaction for nicotine patch assignment; however, graphical exploration found that there was a significant interaction for nicotine patch assignment and participants who reported smoking between 100 and 250 cigarettes a week at baseline (Supporting information, Fig. S1); in these women, assignment to nicotine patch was associated with having smoked fewer cigarettes in the previous 7 days.

## Discussion

Our findings show that women prescribed nicotine patches but who also admitted smoking had similar cotinine concentrations to those generated when they only smoked. These women also reported smoking less and had lower expired air CO readings than when they smoked prior to their quit attempt. In comparison, smokers issued with placebo patches had lower cotinine concentrations than when smoking; they also showed reductions in numbers of cigarettes smoked and expired CO concentrations. Our results also indicate that women who smoke and use nicotine patches smoke less later in pregnancy.

A limitation to our study is that, while we know that women included in this study were prescribed nicotine patches, we have very limited information about how much these were used. However, as study measurements at 2‐ and 8‐week follow up were taken with the intention of personalizing the nicotine doses which women received from patches, it seems very likely that women who attended these appointments were still using these. Furthermore, the SNIPP trial also reports (where adherence data exists) that the median self‐reported adherence rate was 85% 17.

Another possible limitation concerns the validity of women's reports of smoking or not smoking during the week prior to having 2‐ and 8‐week measurements taken. In SNIPP, women were defined as smokers if they had reported any smoking in the week prior to a study visit, and this was validated by an expired CO reading. However, expired air CO can only reliably validate smoking status during the previous 6 hours 25 and, although some women may have over‐ or underestimated the number of cigarettes smoked in the previous week, we could only accurately quantify tobacco smoke exposure in the 6 hours prior to CO measurement. Nevertheless, this could only have had a major impact on findings if women generally under‐reported their smoking during the week prior to follow‐up appointments and, in the 6 hours before follow‐up appointments, tried to smoke less than they had reported. It seems unlikely that trial participants would do this before attending a nicotine patch dose‐titration appointment.

A strength of this study is that the data were obtained as part of a well‐conducted randomized controlled trial and included reported smoking behaviour with concurrent CO and cotinine estimation at several time‐points. To our knowledge, there has been no previous study that has investigated smoking behaviour and CO exposure from concurrent use of nicotine patches and smoking in pregnancy. Hence, we believe this makes an original contribution to the field. Another strength is that comparisons are based on ‘within‐participant’ measurements; this means that inter‐participant variations are very unlikely to explain study findings. Indeed, with this study design one would only expect findings to be affected by characteristics of women which were prone to change between baseline and follow‐up. Women's nicotine metabolic rates (NMR) increase as pregnancy progresses, and these would be expected to affect their plasma nicotine concentrations and so, potentially, also their cravings and intensity of smoking 13, 21. However, any effect would seem to be marginal as, even in the placebo group, women reported smoking fewer cigarettes. Also, as pregnancy‐related NMR acceleration is generally complete by the end of the first trimester and women's mean gestation at baseline was ~13 weeks, there may have been little scope for this factor to have any influence. It seems likely, therefore, that the differences reported reflect differences in smoking behaviour and not changes in women's physiology during pregnancy.

Our study informs about cotinine concentrations in pregnant women who use nicotine patches but are not abstinent from smoking, and show that cotinine concentrations in such women were no higher than when they were smoking. Additionally, women included in this study had simultaneous and statistically significant reductions in their cigarette use, validated by a reduction in expired CO. This suggests that when pregnant women use nicotine patches and smoke, they smoke less than they would if they were not using nicotine patches. This is important, as it could influence how women are advised to use NRT in pregnancy, i.e. encouraged to continue using NRT despite a relapse.

We are unaware of any previous studies measuring cotinine or CO in smokers who concurrently use NRT during pregnancy. A systematic review and meta‐analysis that aimed to identify and describe studies which report nicotine or cotinine concentrations in pregnant women when smoking, and subsequently when abstinent from smoking and using NRT, concluded that among pregnant women who quit smoking, standard‐dose NRT generates lower nicotine exposure than smoking 14. The meta‐analysis compared cotinine exposures when pregnant women smoke with those when they use NRT and found that concentrations were, on average, 75.3 ng/ml lower when abstinent and using NRT than when the same women smoked 14. In SNIPP, salivary cotinine concentrations at baseline (when smoking) were compared to cotinine concentrations at 1 month in women who had stopped smoking but were using nicotine patches. Cotinine concentrations were 98.5 ng/ml while smoking, but only 62.8 ng/ml while using nicotine patches 17. In our study we found that women who were assigned the placebo patch but admitted to smoking also exhibited reduced cotinine concentrations compared to those when smoking alone.

Most studies in the above review used lower nicotine doses than were used by participants in this paper's analyses; other than SNIPP, studies used standard rather than higher doses of nicotine, and these delivered no more than 15 mg cotinine in 16 hours or the 24‐hour equivalent 14. Thus, when pregnant smokers become abstinent and adhere with such ‘standard’ doses of NRT they are, on average, exposed to less nicotine than from smoking 14. In SNIPP, patch doses were adjusted according to the previous saliva cotinine determination to optimize the nicotine substitution leading to somewhat higher mean nicotine doses than usual (18 mg/day, SD = 6.8). It is expected that the dose adjustment would improve nicotine substitution, thus it is possible that women assigned nicotine patches in the 8‐week sample would have higher cotinine concentrations than they had at baseline. Despite this adjustment, there was no significant difference in cotinine concentrations in women who were assigned nicotine patches and admitted to smoking compared to those when smoking alone. This also suggests that smoking and using nicotine patches of ‘standard’ doses may lead to lower cotinine concentrations during pregnancy than smoking alone, prior to pregnancy.

Our findings provide the first data we are aware of which quantifies pregnant women's smoking behaviour when using nicotine patches, and this suggests that when pregnant women use nicotine patches as part of a quit attempt, but also smoke, they smoke less than they did before the quit attempt started. This means that their exposure to the toxic products of burnt tobacco is reduced. A possible reason for this is that women who continue to smoke when using nicotine patches obtain nicotine from both patches and tobacco, and nicotine delivered from patches reduces women's cravings such that they feel less need to ‘top up’ concentrations of nicotine in their body fluids through smoking. This suggests that clinicians can reassure women that it is alright to smoke and use nicotine patches if, ultimately, they are trying for abstinence.

## Conclusions

In conclusion, despite having similar cotinine exposure to that from cigarette smoking, pregnant women who use nicotine patches and smoke, smoke less and exhale less CO, so their exposure to other tobacco smoke toxins is also likely to be lower.

## Declaration of interests

R.C., T.C. and J.L.‐B.: none to declare. I.B. received honoraria for consulting and lectures from Pfizer Ltd.

## Supporting information


**Table S1** Baseline to 8‐weeks after the quit date ‘within‐participant’ differences in indicators of smoking intensity in pregnant smokers by treatment group, with a significance test for interaction with nicotine patch
**Figure S1** Graph to show interaction of nicotine patches on cigarettes smoked at 2‐weeks with increasing number of cigarettes smoked at baseline. The shaded area represents the 95% confidence intervals. As the shaded area for number of cigarettes smoked between 100–250, is below 0, there is a significant interaction of nicotine patches for a reduction of cigarettes smoked at 8‐weeks in women that smoked between 100–250 cigarettes in the week prior to baseline compared with placebo.
