# Supplementary material for: Saliva cotinine concentrations in pregnant women who smoke and use nicotine patches
Source: Addiction. 2019 Jun 30;114(9):1651–8. doi: 10.1111/add.14662 (PMC6771792; doi:10.1111/add.14662)
Supplement: Supplementary file 1 — Table S1 Baseline to 8‐weeks after the quit date ‘within‐participant’ differences in indicators of smoking intensity in pregnant smokers by treatment group, with a significance test for interaction with nicotine patch Figure S1 Graph to show interaction of nicotine patches on cigarettes smoked at 2‐weeks with increasing number of cigarettes smoked at baseline. The shaded area represents the 95% confidence intervals. As the shaded area for number of cigarettes smoked between 100–250, is below 0, there is a significant interaction of nicotine patches for a reduction of cigarettes smoked at 8‐weeks in women that smoked between 100–250 cigarettes in the week prior to baseline compared with placebo. [file ADD-114-1651-s001.docx]

Saliva cotinine concentrations in pregnant women who smoke and use nicotine patches – supporting tables

| **Table 2.** Baseline to 8-weeks after the quit date ‘within-participant’ differences in indicators of smoking intensity in pregnant smokers by treatment group, with a significance test for interaction with nicotine patch. | | | | | | | | | |
| --- | --- | --- | --- | --- | --- | --- | --- | --- | --- |
|  | **Nicotine Patch (n=86)** | | | | **Placebo Patch (n=69)** | | | | Interaction p-value^3^ |
| Characteristic | Baseline mean (SD) | 8-weeks after quit date mean (SD) | Mean difference (95% CI) | p-value^1^ | Baseline mean (SD) | 8-weeks after quit date mean (SD) | Mean difference (95% CI) | p-value^2^ |  |
| Saliva cotinineᶧ (ng/ml) | 116.35 | 98.24 | 0.85 (0.71 to 1.00) | 0.055 | 118.13 | 87.11 | 0.74  (0.61 to 0.89) | 0.002 | 0.874 |
| Expired air carbon monoxide (ppm) | 11.1  (6.3) | 7.8  (5.7) | -3.3  (-4.6 to -1.9) | <0.001 | 12.5  (7.8) | 10.1  (10.7) | -2.4  (-5.0 to 0.3) | 0.077 | 0.844 |
| FTCQ-12^4^ | 32.60  (8.30) | 30.90  (7.56) | -1.69  (-3.58 to 0.20) | 0.079 | 35.16  (7.99) | 31.59  (7.02) | -3.57  (-5.65 to -1.48) | 0.001 | 0.623 |
| Number of cigarettes smoked per day | 12  (8) | 6  (4) | -7  (-8 to -5) | <0.001 | 12  (6) | 7  (6) | -5  (-6 to -3) | <0.001 | 0.132 |
| Number of cigarettes partner smoked per day | 16  (7) | 14  (6) | -2  (-3 to -1) | 0.001 | 15  (7) | 14  (7) | -2  (-3 to 0) | 0.039 | 0.671 |
| Paired t-tests were used to compare differences at baseline and 8-weeks after the quit date. A linear model was used to test for an interaction of nicotine patch between baseline and 8-weeks. | | | | | | | | | |
| ^1^P-value for the difference between indicators of smoking intensity between baseline and 8-weeks, in the nicotine patch group | | | | | | | | | |
| ^2^P-value for the difference between indicators of smoking intensity between baseline and 8-weeks, in the placebo patch group | | | | | | | | | |
| ^3^P-value for interaction of nicotine patch with indicators of smoking intensity at baseline compared with at 8-weeks after the quit date | | | | | | | | | |
| ^4^FTCQ -12– French Tobacco Craving Questionnaire score  ᶧBack transformed saliva cotinine data. Means represent geometric means. Mean difference presented as ratio of geometric means. | | | | | | | | | |

**Figure S1** Graph to show interaction of nicotine patches on cigarettes smoked at 2-weeks with increasing number of cigarettes smoked at baseline. The shaded area represents the 95% confidence intervals. As the shaded area for number of cigarettes smoked between 100-250, is below 0, there is a significant interaction of nicotine patches for a reduction of cigarettes smoked at 8-weeks in women that smoked between 100-250 cigarettes in the week prior to baseline compared with placebo.
